# Life’s Maxims

**Published:** 1860-10

**Authors:** 


### LIFE'S MAXIMS.

NEAR by the little cottage on the banks of the Hudson, among the dozen dead from the burning of the "Henry Clay," there was one form which attracted attention above all others; it was that of a tall, old man, who had already lived beyond his three-score and ten; there was in his features a dignity in death, which showed without information, that he had been a man of mark in his day. On opening his pocket-book there was written the honored name of

STEPHEN ALLEN, and among the papers, there was found a printed scrap, dingy and soiled, almost worn out with the frequent foldings and unfoldings, showing very clearly that it had been perused often and long for counsel and guidance; its principles and its precepts embody the secret of a long life; of a healthful, useful, and honorable old age. We lived near by at the time, and the whole scene has left a life-long impression. The paper was entitled:

### THE MAXIMS OF LIFE;

#### OR, HOW TO BE HAPPY.

Keep good company or none.

Never be idle.

If your hands can not be usefully employed, attend to the cultivation of your mind.

Live up to your engagements.

Keep your own secrets, if you have any.

When you speak to a person, look him in the face.

Good character is above all things else.

Your character can not be essentially injured except by your own acts.

If any one speaks evil of you, let your life be such that none will believe him.

Drink no kind of intoxicating liquors.

Ever live (misfortunes excepted) within your income.

When you retire to bed, think over what you have been doing during the day.

Make no haste to be rich, if you would prosper.

Small and steady gains give competency, with tranquillity of mind.

Never play at any game of chance.

Avoid temptation, through fear you may not withstand it.

Earn money before you spend it.

Never run into debt, unless you see a way to get out again.

Never borrow, if you can possibly avoid it.

Never speak evil of any one.

Be just before you are generous.

Keep yourself innocent, if you would be happy.

Save when you are young, to spend when you are old.

